# Functional properties of dorsolateral prefrontal cortex to primary motor cortex connectivity: a dual-site TMS study

**DOI:** 10.3389/fnhum.2026.1776794

**Published:** 2026-03-11

**Authors:** Xiang-Ming Lin, Yi-Shan Xue, Yu-Han Liu, Rui Hong, Wan-Rong Xu, Tian-Cheng Li, Jia-Wei Jiang, Ying-Rong Liu, Ying Li, Ben-Guo Wang

**Affiliations:** 1 Rehabilitation Medicine Department of The Second Affiliated Hospital, School of Medicine, The Chinese University of Hong Kong, Shenzhen and Longgang District People's Hospital of Shenzhen, Shenzhen, Guangdong, China; 2 Gannan Medical University, Ganzhou, Jiangxi, China; 3 Hubei University of Medicine, Shiyan, Hubei, China

**Keywords:** dorsolateral prefrontal cortex, dual-site transcranial magnetic stimulation, intrahemispheric connectivity, motor evoked potential amplitude, primary motor cortex

## Abstract

**Background:**

The dorsolateral prefrontal cortex (DLPFC) plays a crucial role in cognitive-motor integration through its top-down regulation of the primary motor cortex (M1). However, the functional lateralization of the left and right DLPFC and the differences between intra-hemispheric and inter-hemispheric regulation of M1, particularly in populations with brain injury, remain controversial and insufficiently studied.

**Objective:**

This study aimed to systematically achieve the following four objectives using a dual-site paired-pulse transcranial magnetic stimulation (TMS) technique: (1) to evaluate the integrated regulatory effects of bilateral DLPFC on M1; (2) to compare the differences in regulatory effects between ipsilateral and contralateral DLPFC; (3) to analyze the impact of functional lateralization of the left and right DLPFC on their regulation of M1; (4) to investigate the effects of brain injury on the DLPFC-M1 regulatory pathway by comparing healthy participants and stroke patients.

**Methods:**

A total of 30 right-handed participants were enrolled, including 20 stroke patients in the recovery phase (divided into left and right lesion groups) and 10 healthy volunteers. These three participant groups were tested under conditions that varied the targeted motor cortex (M1) side, yielding four key experimental conditions for analysis. Accordingly, a paired-pulse TMS paradigm was employed. Following a conditioning stimulus (CS) applied to the left or right DLPFC, a test stimulus (TS) was delivered to the ipsilateral or contralateral M1 after an inter-stimulus interval of 20 ms. The amplitude of the motor evoked potential (MEP) was recorded.

**Results:**

In experiments targeting the left M1, both the healthy group (Experiment 1) and the patient group (Experiment 3) exhibited significant regulatory effects (*χ*^2^ = 12.2, *p* = 0.002; *χ*^2^ = 9.6, *p* = 0.008). Post-hoc analysis revealed that, compared to baseline, both ipsilateral DLPFC (*p* = 0.011; *p* = 0.022) and contralateral DLPFC (*p* = 0.005; *p* = 0.022) significantly enhanced M1 excitability, with no significant difference between the two (*p* = 1.000). However, in experiments targeting the right M1 across all groups (Experiments 2 and 4), no significant regulatory effect of DLPFC was observed (*χ*^2^ = 0.2, *p* = 0.905).

**Conclusion:**

This study confirms that, at rest, the bilateral DLPFC exerts a stable and non-specific facilitatory regulation on the left M1. This effect persists in the affected M1 of stroke patients, suggesting plasticity in the relevant pathways after injury. The negative findings for the right M1 reveal a lateralization characteristic in DLPFC-M1 regulation. These results provide an important basis for elucidating the physiological mechanisms of cognitive-motor circuits and for developing targeted neurorehabilitation strategies.

## 1 Introduction

The primary motor cortex (M1) serves as the final output pathway for voluntary movement and is a well-established core target in the field of neuromodulation for treating motor disorders (Bai et al., 2022; Lefaucheur et al., 2020). However, interventions solely targeting M1 often fall short in addressing concomitant cognitive-motor integration deficits following brain injury (Xu et al., 2023; Lin et al., 2025). In recent years, the dorsolateral prefrontal cortex (DLPFC), a key brain region for higher-order cognitive functions such as decision-making and attention, has demonstrated unique value in the field of neurorehabilitation (Brown et al., 2019). Through its top-down regulation of the motor cortex, the DLPFC can compensate for the limitations of targeting motor areas alone. This mechanism is grounded in the functional property of the DLPFC to exert neural influence over the motor cortex (including M1) via both ipsilateral and contralateral polysynaptic pathways (Selemon and Goldman-Rakic, 1988; Gabbott et al., 2005; Yeterian et al., 2012). These connections enable the DLPFC to mediate advanced cognitive processes within motor control, including executive function (Szameitat et al., 2002), response selection (Rowe et al., 2000), initiation (Jahanshahi et al., 1995), and inhibition (Jahanshahi et al., 2015), thereby forming a comprehensive cognitive-motor regulatory network. Consequently, contemporary neurorehabilitation paradigms are gradually shifting from singular M1 modulation toward novel strategies involving coordinated intervention on dual DLPFC-M1 targets. This integrative therapy, based on neural circuit mechanisms, holds promise not only for optimizing motor output but also for opening new therapeutic dimensions in promoting post-injury neural reorganization by reshaping the cognitive-motor interface (Xu et al., 2024).

Investigating the modulatory effects of the DLPFC on M1 is of significant importance for unveiling the neural mechanisms underlying cognitive and motor integration. The decline in motor and cognitive functions associated with aging or neurological disorders (e.g., Parkinson’s disease, Alzheimer’s disease, and stroke) is often accompanied by impairments in the connectivity of related brain networks (Michely et al., 2018; King et al., 2018). Therefore, elucidating the functional connectivity properties of the DLPFC-M1 circuit not only contributes to a deeper understanding of the operational mechanisms of the brain’s motor control network but also provides crucial neurophysiological evidence for the clinical treatment of cognitive-motor disorders. Currently, findings regarding DLPFC modulation of M1 remain controversial: some studies have reported inhibitory effects (Wang et al., 2020), others have observed facilitatory effects (Cao et al., 2022), and some have found no significant influence (Brown et al., 2019). Most of these studies have examined intra-hemispheric or inter-hemispheric connections in isolation, failing to systematically compare the differences between them. Furthermore, the left and right DLPFC are believed to possess distinct functional properties—the left DLPFC may be more involved in motor execution and control (Jin et al., 2019), whereas the right DLPFC is more engaged in motor inhibition (Pierrot-Deseilligny et al., 2005). However, whether this functional lateralization leads to differential modulatory effects on M1 requires further investigation. Additionally, existing research has primarily been conducted in healthy populations, with validation in brain injury models being insufficient. In particular, whether the regulation of the affected M1 by the DLPFC changes after stroke remains to be thoroughly investigated.

To this end, this study employed a dual-site paired-pulse transcranial magnetic stimulation (TMS) technique to systematically assess the modulatory effects of the DLPFC on M1 through four independent experiments. This technique allows for the effective probing of immediate modulatory effects of the DLPFC on M1 excitability by precisely controlling the temporal interval and intensity between the conditioning stimulus (CS) and the test stimulus (TS) (Rothwell, 2011; Hallett et al., 2017). The study first evaluated the integrated regulatory effects of bilateral DLPFC on M1. It then compared the differences in regulatory effects between ipsilateral and contralateral DLPFC while also analyzing the impact of functional lateralization of the left and right DLPFC on their modulation of M1. We hypothesized that, given the proposed dominant role of the left DLPFC in motor execution, it would exert a stronger facilitatory effect on M1, even across hemispheres and in the presence of stroke. More importantly, this study aimed to extend these basic findings to the clinical context after brain injury. Although stroke lesions typically do not directly damage the DLPFC-M1 circuitry, sensorimotor integration deficits are common and persistent post-stroke, severely hampering functional recovery (Edwards et al., 2019). Given the crucial role of the DLPFC in integrating somatosensory information to guide motor control (Brown et al., 2018), studies have attempted to improve sensorimotor integration by stimulating the DLPFC (Lefaucheur et al., 2020). However, against the backdrop of widespread network reorganization following stroke, it remains insufficiently studied whether this regulatory pathway retains its functional efficacy and can continue to serve as an effective intervention target. Therefore, by comparing healthy participants and stroke patients, this study investigated the influence of brain injury on the DLPFC-M1 regulatory pathway, which will help elucidate the plasticity of the cognitive-motor network after stroke and provide key physiological evidence for developing novel rehabilitation strategies based on precise neuromodulation.

## 2 Materials and methods

### 2.1 Participants

All participants were right-handers as determined by the Edinburgh Handedness Inventory (10-item version, mean laterality quotient = 86 ± 11.2) (Oldfield, 1971). The patient group comprised 20 stroke patients in the recovery phase, whose diagnosis complied with the Chinese Guidelines for the Diagnosis and Treatment of Acute Ischemic Stroke and met the internationally recognized AHA/ASA management standards (Powers et al., 2019). Based on cranial CT results, patients were divided into two groups: a left hemisphere lesion group (*n* = 10, mean age 56.5 ± 4.8 years) and a right hemisphere lesion group (*n* = 10, mean age 55.8 ± 5.1 years). Additionally, 10 healthy volunteers with no history of neurological disorders (mean age 52.2 ± 3.1 years) were recruited. All participants were screened for TMS contraindications and provided written informed consent. The study was approved by the Ethics Committee of Shenzhen Longgang District People’s Hospital (Approval No. AP2025128).

The study sample comprised three distinct participant groups: 10 healthy controls, 10 patients with left-hemisphere stroke, and 10 patients with right-hemisphere stroke. The experimental design was built upon two factors: participant group (healthy, left-stroke, right-stroke) and the laterality of the M1 stimulation target (ipsilateral or contralateral to the conditioned DLPFC). The combination of these factors defined the four core experimental conditions, corresponding to the following four experimental configurations: (1) Healthy-Left M1 group: 10 healthy volunteers, to observe the effects of bilateral DLPFC on their left M1; (2) Healthy-Right M1 group: 10 healthy volunteers, to observe the effects of bilateral DLPFC on their right M1; (3) Patient-Left M1 group: 10 stroke patients with left hemisphere lesions, to observe the effects of bilateral DLPFC on their left M1; (4) Patient-Right M1 group: 10 stroke patients with right hemisphere lesions, to observe the effects of bilateral DLPFC on their right M1.

### 2.2 Motor evoked potential (MEP) recording

**Figure 1:**
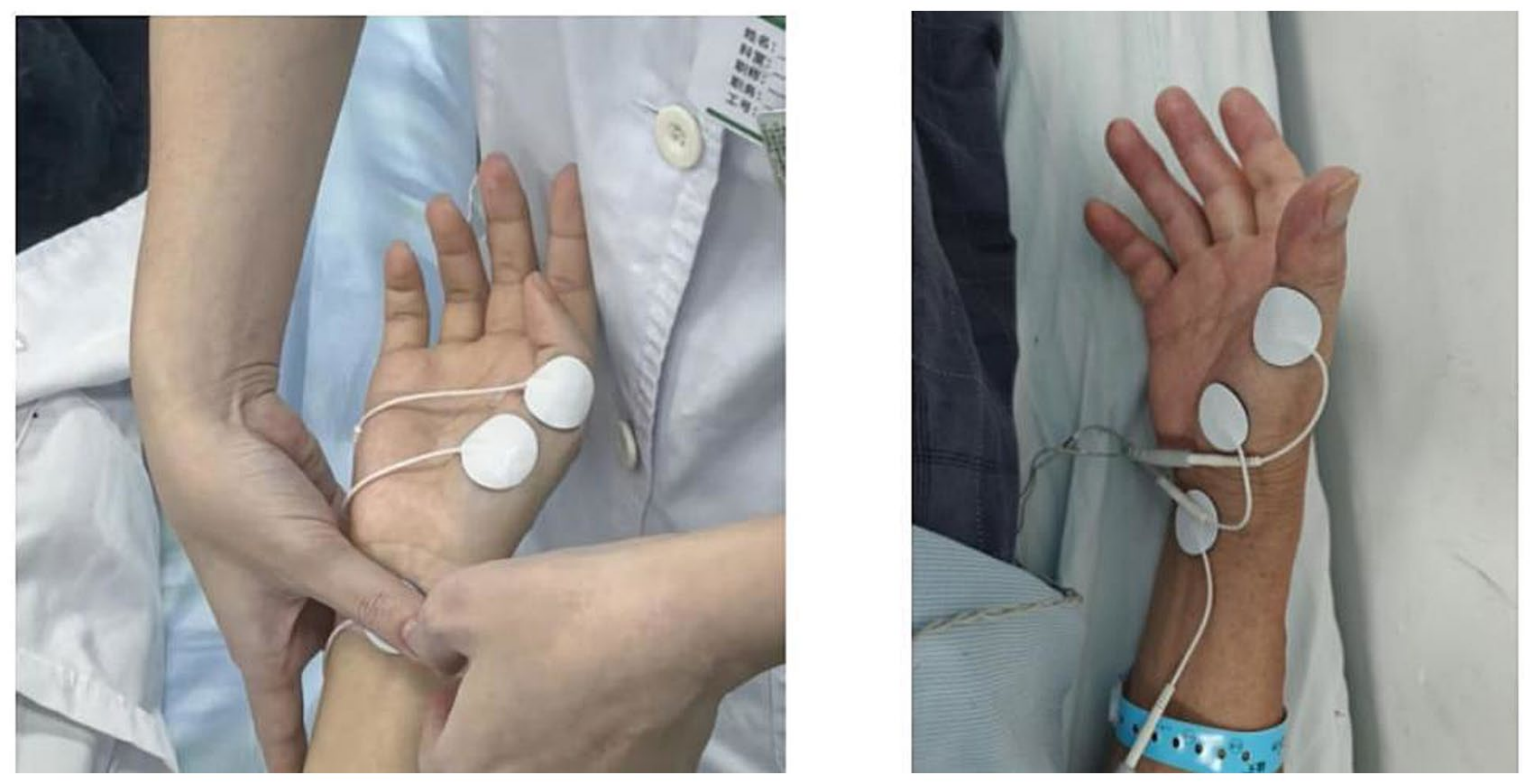
Schematic diagram of surface electrode placement.

MEPs were recorded using the integrated MEP (electromyography, EMG) module of a transcranial magnetic stimulation device (model: MagTD 80-A; Yiruide, Wuhan, China). All participants were tested in a supine position under quiet and relaxed conditions. The recording electrode was placed over the belly of the abductor pollicis brevis (APB) muscle of the target arm, the reference electrode was positioned on the tendon of the same muscle, and the ground electrode was placed at the wrist (Groppa et al., 2012). This placement conforms to guideline recommendations and is optimal for recording MEPs from the hand muscles innervated by M1 (Lefaucheur et al., 2020) (Figure 1).

### 2.3 Transcranial magnetic stimulation (TMS)

**Figure 2:**
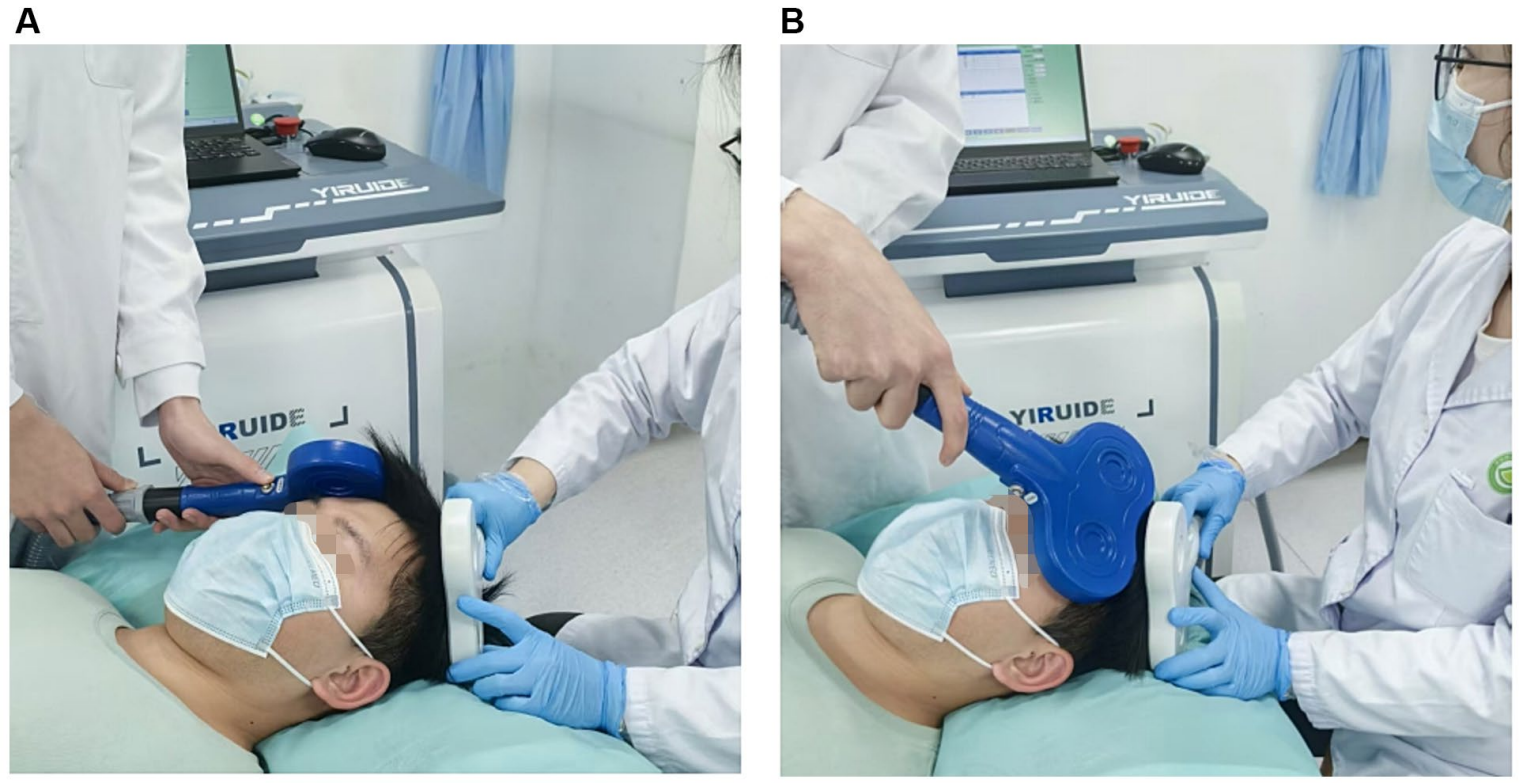
Schematic illustration of dual-coil operation during paired-pulse TMS: **(A)** Ipsilateral stimulation; **(B)** Contralateral stimulation.

This study employed a dual-site paired-pulse TMS paradigm using a transcranial magnetic stimulation device (model: MagTD 80-A; Yiruide, Wuhan, China) and two figure-of-eight coils. The stimulator delivered a biphasic pulse waveform. All participants received stimulation in a quiet and relaxed supine position. The resting motor threshold (RMT) was defined according to international standards as the lowest stimulus intensity that could elicit MEPs of at least 50 μV in the contralateral target muscle in at least 5 out of 10 consecutive trials while the participant’s hand muscles remained relaxed (Rossini et al., 2015). The intensity of the TS was set at 120% RMT, an intensity widely used in previous studies to reliably evoke MEPs and assess corticospinal tract excitability (Lefaucheur et al., 2020). Referring to the study by Brown et al. (2019), the effects of CS intensities of 80 and 120% RMT over the DLPFC on bilateral M1 were similar. To ensure that the CS over the DLPFC could elicit an effective physiological effect while considering patient tolerance and operational safety, the CS intensity over the DLPFC was ultimately set at 100% RMT in this study. The intensities of TS and CS were determined independently for the left and right hemispheres. The CS coil was placed over the left or right DLPFC [Brodmann area 46 (Rowe et al., 2002)]. The TS was applied to the ipsilateral or contralateral hotspot of M1 20 ms after the CS delivery. This paired-pulse paradigm was physically implemented using two independent figure-of-eight coils (wing diameter: 70 mm), both driven by the dual-channel output of the MagTD 80-A TMS system. One coil was positioned over the target DLPFC to deliver the CS, and the other over the target M1 to deliver the TS. For intrahemispheric conditions, an experienced experimenter carefully held both coils at appropriate angles to ensure precise targeting over the respective cortical hotspots while preventing physical interference (Figure 2). The M1 stimulating coil was positioned to induce a posterior–anterior directed current; the CS coil over the DLPFC was positioned to induce an anterior–posterior directed current. According to the study by Ni et al. (2009), different CS current directions over the DLPFC had similar effects on M1.

Taking Experiment 1 (Healthy-Left M1 group) as an example: First, the RMT for the left hemisphere targeting the designated muscle (e.g., the right abductor pollicis brevis, APB) was determined for each participant. Subsequently, each participant received three stimulation conditions in a randomized order: (1) Single-pulse condition: TS applied to the left M1 alone; (2) Dual-pulse condition (Left DLPFC): CS applied to the left DLPFC followed by a TS to the left M1 after a 20-ms interval; (3) Dual-pulse condition (Right DLPFC): CS applied to the right DLPFC followed by a TS to the left M1 after a 20-ms interval. This paired-pulse paradigm is based on the established physiological principle that, when the interstimulus interval (ISI) is set within a range of approximately 8–40 ms, a suprathreshold CS applied to a conditioning site (such as the DLPFC) can produce a net facilitatory effect, resulting in an increase in the amplitude of MEPs evoked by a subsequent TS in the contralateral resting target muscle. Therefore, our selection of a 20 ms ISI, situated within this classical facilitatory window, was intended to specifically probe the excitatory influence of the DLPFC on M1 excitability (Lefaucheur et al., 2020; Brown et al., 2019).

**Figure 3:**
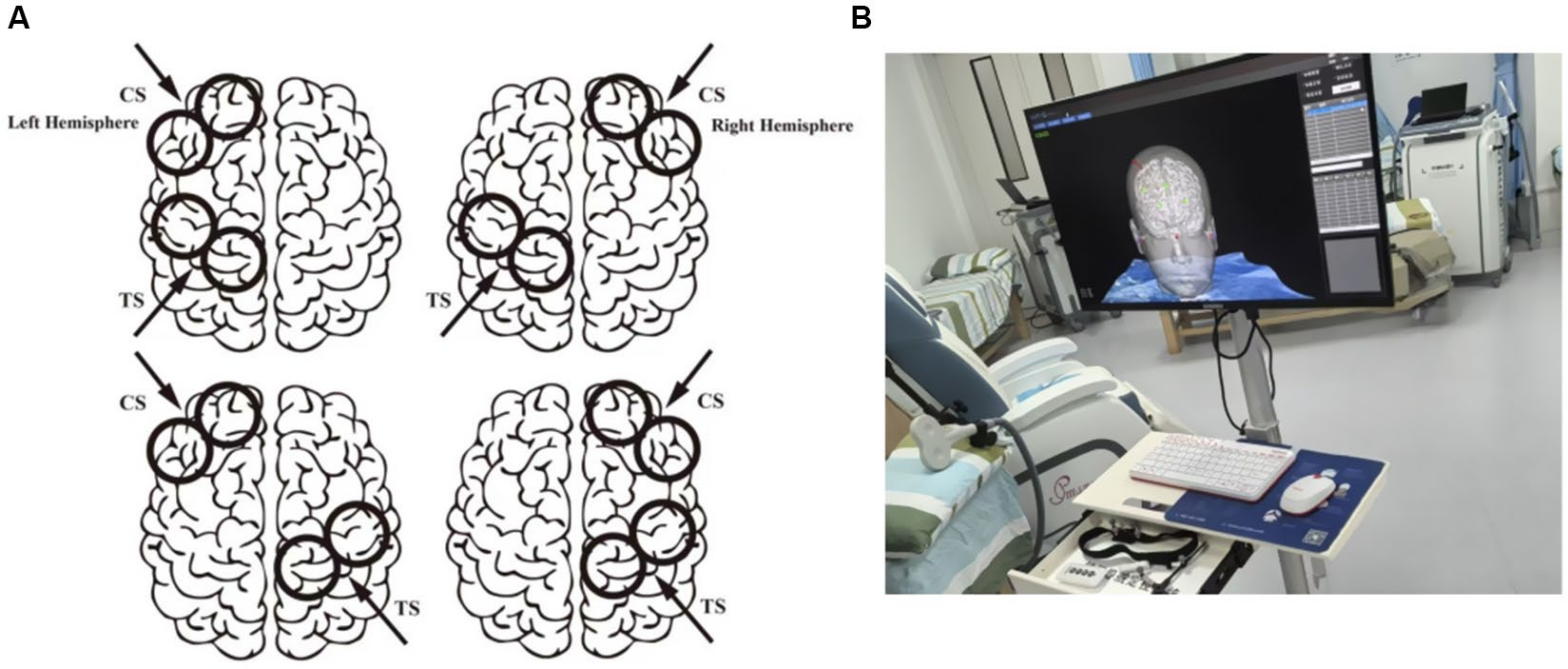
Experimental protocol and neuronavigation for dual-site TMS. **(A)** Schematic diagram of the paired-pulse TMS stimulation protocol for DLPFC–M1 connectivity. CS: Conditioning stimulus applied to the DLPFC; TS: test stimulus applied to M1. The illustration shows different stimulation conditions for DLPFC–M1 combinations (ipsilateral and contralateral); **(B)** Individualized neuronavigation for TMS target localization.

The peak-to-peak amplitude of each MEP was automatically identified and extracted by the device’s integrated software within a post-stimulus window of 20–50 ms, after excluding segments with excessive noise. For the single-pulse baseline, a minimum of 10 valid trials were collected. A trial was included only if it met the following criteria: (1) no discernible muscle pre-activation (pre-TS background EMG root-mean-square < 15 μV), and (2) a clear MEP with a peak-to-peak amplitude ≥0.05 mV. The mean amplitude of all valid trials defined the baseline MEP. For each dual-pulse condition (CS + TS), a larger block of 30–40 consecutive trials was delivered to obtain a robust response ensemble. Offline, all sweeps were superimposed for visual inspection. The primary quality control was based on waveform consistency: trials with excessive noise or artifacts that markedly deviated from the common response pattern were excluded. From the remaining trials, a subset of approximately 20–30 waveforms demonstrating high morphological consistency (visually assessed by overlap and stable onset latency) was identified. The final MEP amplitude for each dual-pulse condition was calculated as the mean of all trials within this consistent subset. To ensure data stability, this entire acquisition process was repeated three times for each condition. The mean of the three average values was taken as the final MEP amplitude for that condition for subsequent statistical analysis. To avoid interference between different conditions, the stimulation for each condition (e.g., left and right DLPFC) was conducted at the same time point on separate experimental days. Within the same experimental day, the interval between consecutive TMS stimuli (from the start of one stimulus to the start of the next) was fixed at 5 s, and different stimulation conditions were presented in a randomized order. Experiments 2, 3, and 4 followed the same experimental logic, with the stimulation targets (left/right DLPFC, left/right M1) adjusted according to the group objectives (Figure 3A).

To ensure the reliability of MEP signals, stringent quality control was implemented during the acquisition phase: the electrophysiological signals of the target muscle were monitored in real-time to exclude invalid trials with unclear MEP waveforms or excessive baseline drift due to inadequate muscle relaxation. In some patients with severe corticospinal tract damage, no reproducible MEPs could be elicited even at the maximum stimulation intensity, which was regarded as an inability to evoke MEPs from that side. Consequently, data from 5 patients (3 with left-sided lesions, 2 with right-sided lesions) were excluded. Supplementary recruitment was conducted to ensure that the final valid sample size for each group met the predetermined plan, thereby ensuring the statistical power of subsequent analyses.

### 2.4 Conditioning stimulus site

Based on the specific responses validated in previous studies, Brodmann area 46 was defined as the target location for the DLPFC in this study, with Talairach coordinates of ±40, 28, 30 (Mylius et al., 2013). The precise location of the DLPFC for each participant was individually determined and navigated based on their individual T1-weighted structural magnetic resonance imaging (MRI) scans, using the NHT-NV300 TMS positioning medical imaging processing software (Brain Navigation Technology Co., Ltd., Guangzhou, China) (Figure 3B). In contrast, the M1 hotspot was functionally localized for each participant prior to the main experiment. This was achieved by systematically mapping the contralateral motor cortex with single-pulse TMS at a suprathreshold intensity to locate the scalp position that consistently elicited the largest MEP amplitude in the target abductor pollicis brevis muscle. This functional ‘hotspot’ was then marked and recorded within the same neuronavigation system for precise coil repositioning throughout the experiment.

### 2.5 Data analysis

All MEP amplitude data were processed using SPSS software (version 26.0). As the data were confirmed to be non-normally distributed by the Shapiro–Wilk test, they are presented as median (interquartile range). Friedman tests were used to analyze differences in MEP amplitudes under different stimulation conditions (baseline, left DLPFC → M1, right DLPFC → M1) within each group. Post-hoc pairwise comparisons for all possible combinations of stimulation conditions were further conducted using Wilcoxon signed-rank tests. To control for inflation of type I error due to multiple comparisons, the *p*-values from all pairwise comparisons were adjusted using the Bonferroni correction method.

## 3 Results

### 3.1 Modulatory effects of DLPFC on M1 in healthy participants

**Figure 4:**
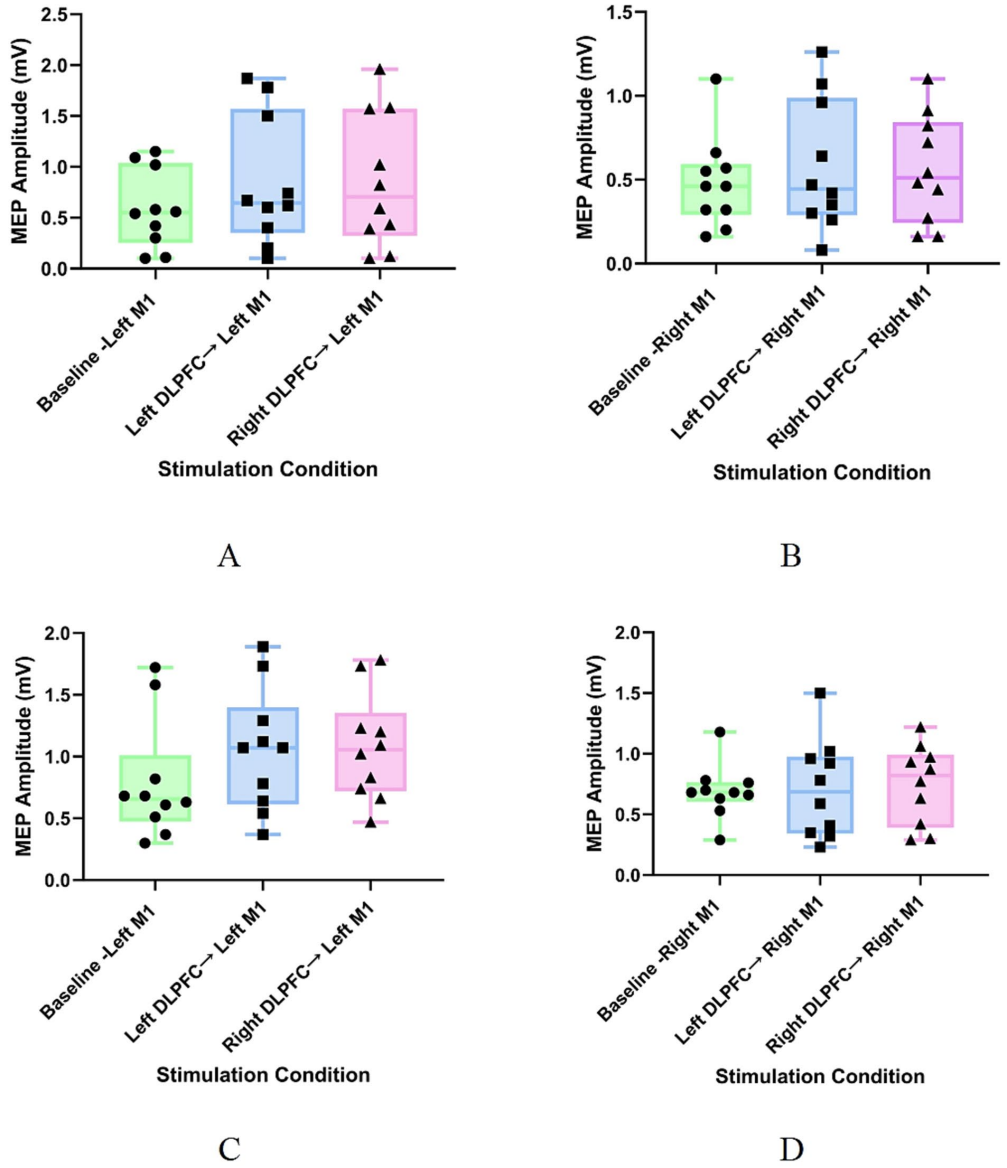
Changes in MEP amplitudes under different stimulation conditions across groups. **(A)** Group 1: Healthy control – left M1 group; **(B)** Group 2: Healthy control – right M1 group; **(C)** Group 3: Patient – left M1 group; **(D)** Group 4: Patient – right M1 group. Baseline: TS applied to M1 alone. Baseline: TS applied to M1 alone; left DLPFC → M1: CS applied to the left DLPFC followed by a TS to M1; right DLPFC → M1: CS applied to the right DLPFC followed by a TS to M1.

In healthy participants, the Friedman test for Experiment 1 (Healthy-Left M1 group) showed a significant effect of different stimulation conditions on MEP amplitudes of the left M1 (*χ*^2^ = 12.2, *p* = 0.002) (Table 1; Figure 4A). Post-hoc analysis (with Bonferroni correction) indicated that, compared to the single-pulse condition, both the ipsilateral (left) DLPFC conditioning stimulus (*p* = 0.011) and the contralateral (right) DLPFC conditioning stimulus (*p* = 0.005) significantly enhanced the excitability of the left M1. There was no significant difference between the modulatory effects of the ipsilateral and contralateral DLPFC (*p* = 1.000) (Table 2). However, in Experiment 2 (Healthy-Right M1 group), no significant effect of different stimulation conditions on MEP amplitudes of the right M1 was observed (*χ*^2^ = 0.2, *p* = 0.905) (Table 1; Figure 4B).

**Table 1:** Comparison of MEP amplitudes under different stimulation conditions in each group.

Group	Baseline	Left DLPFC → M1	Right DLPFC → M1	*χ* ^2^	*p*
Group 1: Healthy Control—Left M1 Group	0.654 (0.477, 0.101)	1.07 (0.618, 1.401)	1.05 (0.722, 1.357)	12.2	0.002*
Group 2: Healthy Control—Right M1 Group	0.682 (0.603, 0.764)	0.682 (0.341, 0.976)	0.820 (0.386, 0.995)	0.2	0.905
Group 3: Patient—Left M1 Group	0.550 (0.255, 1.037)	0.644 (0.351, 1.567)	0.706 (0.321, 1.571)	9.6	0.008*
Group 4: Patient—Right M1 Group	0.460 (0.287, 0.591)	0.448 (0.286, 0.990)	0.512 (0.245, 0.843)	0.2	0.905

MEP amplitudes are reported in millivolts (mV). Data are presented as median (interquartile range). Friedman test was used to compare MEP amplitudes among three stimulation conditions (Baseline, Left DLPFC → M1, Right DLPFC → M1) within each group. *Indicates *p* < 0.05, statistically significant.

**Table 2:** Pairwise comparisons of MEP amplitudes between different stimulation conditions in each group.

Group	Baseline vs. Left DLPFC	Baseline vs. Right DLPFC	Left DLPFC vs. Right DLPFC
Group 1: Healthy Control—Left M1 Group	0.011*	0.005*	1.000
Group 2: Healthy Control—Right M1 Group	0.999	0.960	0.995
Group 3: Patient—Left M1 Group	0.022*	0.022*	1.000
Group 4: Patient—Right M1 Group	0.886	0.912	0.999

Wilcoxon signed-rank test was used for pairwise comparisons, with *p*-values adjusted by Bonferroni correction. *Indicates *p* < 0.05, statistically significant.

### 3.2 Modulatory effects of DLPFC on M1 in stroke patients

In stroke patients, the Friedman test for Experiment 3 (Patient-Left M1 group) showed a significant effect of different stimulation conditions on MEP amplitudes of the affected (left) M1 (*χ*^2^ = 9.6, *p* = 0.008) (Table 1; Figure 4C). Post-hoc analysis (with Bonferroni correction) indicated that, compared to the single-pulse condition, both the ipsilateral (left) DLPFC conditioning stimulus (*p* = 0.022) and the contralateral (right) DLPFC conditioning stimulus (*p* = 0.022) significantly enhanced the excitability of the affected M1. There was no significant difference between the modulatory effects of the ipsilateral and contralateral DLPFC (*p* = 1.000) (Table 2). In contrast, in Experiment 4 (Patient-Right M1 group), no significant effect of different stimulation conditions on MEP amplitudes of the affected (right) M1 was observed (*χ*^2^ = 0.2, *p* = 0.905) (Table 1; Figure 4D).

## 4 Discussion

This study systematically investigated the modulatory effects of the left and right DLPFC on the excitability of ipsilateral and contralateral M1 using a dual-site paired-pulse TMS technique. The results revealed that when targeting the left M1 (Experiments 1 and 3), both ipsilateral and contralateral DLPFC exhibited similar and significant facilitatory effects, with no observed functional lateralization of the left and right DLPFC or pathway-specific (intra-hemispheric/inter-hemispheric) differences. On one hand, regarding the functional specificity of the left and right DLPFC, although extensive literature suggests that the left DLPFC may be more involved in motor execution and control (Rubia et al., 2001), while the right DLPFC is more associated with inhibitory functions (Pierrot-Deseilligny et al., 2005), this study did not observe such clear lateralization under resting-state conditions. This differs from the findings of Hasan et al., who reported a specific facilitatory effect of the left DLPFC on ipsilateral M1 during a behavioral task (Hasan et al., 2013). A possible explanation for this discrepancy lies in the difference in experimental paradigms: their study was conducted in the specific context of a choice reaction task, integrating cognitive-motor demands, whereas the present study measured baseline connectivity at rest. This suggests that DLPFC modulation of M1 may be highly state-dependent, with functional lateralization becoming prominent under complex cognitive demands. In contrast, under basic resting-state conditions, bilateral DLPFC may exert similar, non-specific facilitatory influences on M1 via shared or parallel pathways.

On the other hand, concerning the regulatory pathways (intra-hemispheric versus inter-hemispheric), a key finding of this study is the absence of significant differences in modulatory effects between ipsilateral and contralateral DLPFC → M1 pathways. This contrasts with some studies emphasizing the dominance of intra-hemispheric connectivity (Koch et al., 2007). Given the lack of direct anatomical connections (Miller and Cohen, 2001) and direct white matter fiber tracts (Civardi et al., 2001) between the DLPFC and M1, the neural mechanisms underlying this similarity in ipsilateral and contralateral regulatory effects warrant further investigation. As proposed in the model by Neubert et al. (2010), DLPFC modulation of M1 is likely mediated via a complex polysynaptic network (Xia et al., 2022). This model posits that prefrontal signals may be relayed through common “relay stations,” such as the dorsal premotor cortex or basal ganglia circuits. Our findings provide strong electrophysiological evidence in support of this mechanism, indicating that signals from both intra-hemispheric and inter-hemispheric pathways may ultimately converge within this polysynaptic network, thereby generating similar net excitatory effects at the level of M1.

More importantly, this study extends the aforementioned findings to the context of brain injury. Previous research has predominantly focused on healthy populations (Wang et al., 2020; Cao et al., 2022; Vaseghi et al., 2015), with insufficient attention paid to the circuit functions of the affected M1 following brain injury. Our results demonstrate that in the affected M1 of stroke patients (Experiment 3), a facilitatory effect mediated by bilateral DLPFC, similar to that observed in the healthy control group (Experiment 1), was still present. This finding aligns with and corroborates the observations of Fujiyama et al. (2016) and Verstraelen et al. (2020), who reported that bilateral DLPFC could facilitate contralateral M1 during complex bimanual tasks. Moreover, it further extends the efficacy of such interhemispheric regulation to the resting state in patients during stroke recovery. This indicates that, despite the presence of brain injury, the regulatory pathways from bilateral DLPFC to the affected M1, particularly the inter-hemispheric pathway, retain a degree of integrity and functional viability. This provides important electrophysiological evidence for understanding post-injury network reorganization and for developing novel rehabilitation strategies targeting the DLPFC.

However, in stark contrast to the clear responses observed for the left M1, no significant modulatory effects of DLPFC were observed in any group during experiments targeting the right M1 (Experiments 2 and 4). This lateralized result is thought-provoking. Considering that all participants enrolled in this study were right-handed, the inherent corticospinal tract excitability level and network connectivity strength of the right M1 (typically corresponding to the non-dominant hand) may be intrinsically lower than those of the left M1 (the dominant hemisphere). This is consistent with previous reports of hemispheric asymmetry in motor cortex excitability (Ziemann and Hallett, 2001; Netz et al., 1995). Therefore, the present results may reflect a “dominant hemisphere preference” in DLPFC regulation of M1, indicating that its modulation is more sensitive and effective for the M1 corresponding to the dominant hand. An alternative possible mechanism lies in the functional division of labor between the left and right DLPFC themselves in specific functions such as motor memory and force control, as suggested by the study of Jin et al. (2019). Such functional specificity might lead to different modes or require different conditions (e.g., stimulation parameters) when regulating the non-dominant M1. In addition to different stimulation parameters (e.g., intensity, ISI), different pulse waveforms should also be considered, although the biphasic waveform is the standard configuration for many modern TMS devices and has also been used in previous key studies on DLPFC-M1 connectivity (Brown et al., 2019; Wang et al., 2020), which ensures the direct comparability of our results with the existing literature. However, the direction of current flow of biphasic pulses within the cortex is different from that of monophasic waveforms, which can lead to differential activation of neural populations and interneuronal circuits (Lucarelli et al., 2025; Guidali et al., 2023), thereby potentially modulating the net result of paired-pulse connectivity measurements. Future studies could directly compare the effects of different pulse waveforms on this pathway.

Nevertheless, this study has several limitations. For example, the stimulation parameters employed (e.g., conditioning stimulus intensity and inter-stimulus interval) were relatively uniform. Systematically varying these parameters in future studies could more comprehensively reveal the functional properties of the DLPFC-M1 circuit. Furthermore, the current results were obtained under resting-state conditions; the role of this circuit during motor or cognitive tasks remains to be further explored. Additionally, the sample size was limited and exclusively drawn from a right-handed population, necessitating caution when generalizing the findings to broader populations. Looking ahead, expanding the sample size, incorporating parameter optimization designs across multiple behavioral paradigms, and enrolling more diverse participants will enable a deeper elucidation of the regulatory mechanisms of the DLPFC-M1 pathway and its clinical translational potential.

## 5 Conclusion

In summary, this study, utilizing a dual-site paired-pulse TMS technique, confirmed that under resting-state conditions, bilateral dorsolateral prefrontal cortex exerts significant and similarly potent facilitatory regulation on the left primary motor cortex. This effect is not constrained by intra-hemispheric or inter-hemispheric pathways and is likely mediated by a shared polysynaptic relay network. Importantly, this regulatory pattern persists in the affected M1 of stroke patients, suggesting a degree of plasticity in the relevant pathways after brain injury. This provides a crucial theoretical basis for developing neurorehabilitation strategies based on the DLPFC-M1 circuit. However, the lack of significant regulatory effects on the right M1 suggests that the underlying mechanisms may exhibit lateralization characteristics. Future research should build upon optimizing stimulation parameters, incorporating behavioral tasks, and expanding sample sizes to further unravel the complete functional profile of this circuit.

## Data Availability

The original contributions presented in the study are included in the article/supplementary material, further inquiries can be directed to the corresponding author.
